# Unraveling Drug Resistance in *Mycobacterium leprae*: Exploring Genetic Mutations to Enhance Treatment Strategies for Human Leprosy—A Narrative Review

**DOI:** 10.1155/ijm/7204337

**Published:** 2025-12-11

**Authors:** Gayathri Perera, Maheshi Thilakarathna, Ishani Aluthgamage, Sakuni Sathsarani, Pasan C. Fernando, Sharini Samaranayake, Nazif Ullah, Bhagya Deepachandi

**Affiliations:** ^1^ Department of Life Sciences, Faculty of Science, NSBM Green University, Homagama, Sri Lanka; ^2^ Faculty of Graduate Studies, University of Sri Jayewardenepura, Nugegoda, Sri Lanka, sjp.ac.lk; ^3^ Department of Plant Sciences, Faculty of Science, University of Colombo, Colombo, Sri Lanka, cmb.ac.lk; ^4^ Department of Biotechnology, Abdul Wali Khan University, Mardan, Pakistan, awkum.edu.pk

**Keywords:** drug resistance, gene mutations, leprosy, multidrug therapy, *Mycobacterium leprae*

## Abstract

Leprosy, one of the oldest diseases, is caused by *Mycobacterium leprae* and *Mycobacterium lepromatosis* and continues to pose a significant global public health challenge despite decades of control efforts and the widespread use of multidrug therapy. Clinical manifestations range from tuberculoid to severe lepromatous forms, often accompanied by immune‐mediated inflammatory reactions. The disease exhibits a long incubation period, high infectivity, and complex immune‐mediated pathology, complicating timely diagnosis and management. Although multidrug therapy comprising rifampicin, dapsone, and clofazimine remains the mainstay treatment recommended by the World Health Organization for leprosy and has proven to be highly effective in managing both multibacillary and paucibacillary forms, the treatment outcomes are hindered by drug resistance, adverse drug reactions, and poor adherence. Resistance primarily arises from genetic mutations in drug target genes such as rpoB, folP1, and gyrA, with additional contributions from efflux mechanisms and cell wall impermeability. This narrative review draws upon a comprehensive search of electronic databases to enhance understanding of the genetic mutations associated with drug resistance. It further highlights ongoing research into resistance mechanisms, novel therapeutic options, postexposure prophylaxis, and vaccine development, which are critical for sustaining the effectiveness of multidrug therapy and advancing global leprosy control efforts.

## 1. Introduction

Leprosy (Hansen’s disease) remains a universal public health challenge, despite decades of control attempts. It is caused by *Mycobacterium leprae* or *Mycobacterium lepromatosis* [1]. In 1873, Gerhard Hansen initially discovered *M. leprae*, and in 2001, Cole et al. sequenced its genome [2]. In 2008, Han et al. identified *M. lepromatosis*, originally found in multibacillary (MB) cases in Mexico and subsequently in paucibacillary (PB) forms [3]. Leprosy is a chronic infectious disease characterized by a long incubation period (2–11 years) along with low pathogenicity but high infectivity, making diagnosis and control difficult [1].

After reaching a prevalence of fewer than one case per 10,000 people, in 2000, the World Health Organization (WHO) declared leprosy as a globally eliminated public health problem [4]. This was largely due to the rollout of multidrug therapy (MDT), introduced in 1982 and provided free of charge since 1995 [4, 5]. MDT, consisting of rifampicin (RIF), dapsone (diamino‐diphenyl sulfone [DDS]), and clofazimine (CFZ), has remained the ground treatment for leprosy since its introduction [6].

Despite these efforts, around 200,000 new cases are still reported annually [5]. Prompt diagnosis and treatment are vital, and monitoring drug resistance is key to universal control strategies [7]. Poor treatment adherence significantly contributes to the appearance of drug‐resistant strains, posing a challenge to the efficacy of MDT in leprosy [8, 9]. Drug resistance has emerged in multiple countries with Brazil and India reporting the highest prevalence of drug‐resistant against *M. leprae* [6].

Among the different drugs, RIF resistance is linked to mutations in the *rpoB* gene, with possible involvement of *ctpC* and *ctpI* [6], and resistance to ofloxacin (OFL) and DDS is associated with mutations in *gyrA* and *folP1*, respectively [10]. Additionally, nonsense mutations in the *nth* excision repair gene have been linked to increased sequence diversity and drug resistance [11]. The inability to culture *M. leprae in vitro* complicates resistance studies requiring the use of armadillos or immune‐deficient mice as hosts [12].

To prevent disease spread and for better management of the disease, WHO now recommends single‐dose RIF as postexposure prophylaxis for contacts of leprosy cases [13]. This increases the urgency of resistance monitoring, although drug susceptibility testing remains limited due to the laborious mouse footpad (MFP) assay, which was once regarded as the gold standard [14]. However, this method is labor‐intensive, time‐consuming, and impractical for large‐scale surveillance. As a result, molecular assays based on PCR amplification followed by DNA sequencing are currently considered the “gold standard” for detecting drug resistance [15, 16].

This narrative review synthesized current knowledge on genetic mutations linked to drug resistance in *M. leprae* by conducting a comprehensive literature search across PubMed, Scopus, and Web of Science for articles, emphasizing the urgent need for improved diagnostics, novel therapies, and stronger surveillance systems to combat drug‐resistant strains and reduce disease burden globally.

## 2. Leprosy and Its Causative Agents

Leprosy is a chronic infectious disease mainly affecting the skin and peripheral nerves, with clinical indications shaped by the host’s immune response [17]. It presents along a spectrum from tuberculoid leprosy (TT), a milder form to the severe lepromatous leprosy (LL) [18]. Nerve involvement leads to sensory loss, motor dysfunction, and deformities, generally causing long‐term disability and social stigma [18, 19].

The main causative agent is *M. leprae*, a slow growing, acid‐fast, obligatory intracellular bacterium that prefers colder bodily parts including the skin and nerves [20, 21]. Its persistence and transmission are contributed by its ability to survive in acidic and harsh environments [22]. *M. leprae* causes a prolonged incubation period and a delayed onset of symptoms, with a generation time of around 2 weeks. Disease control and understanding of transmission patterns are further complicated by its resilience to environmental stressors and genetic uniformity. *M. leprae* infects Schwann cells triggering cellular responses that promote bacterial survival. The infection results in skin as dermatological signs, while in nerves it causes demyelination and axonal damage leading to sensory and motor deficits [19].

Latterly discovered, *M. lepromatosis* has been associated with MB leprosy and is notably linked to a stringent form diffuse lepromatous leprosy (DLL) marked by extensive bacterial infiltration [23, 24]. Unlike *M. leprae*, infections caused by *M. lepromatosis* frequently present with systemic symptoms such as fever, lymphadenopathy, and severe skin lesions [25]. This species has been identified in Mexico, Singapore, and Canada, emphasizing its global relevance especially as a primary cause in parts of Mexico [25].

Leprosy is still endemic in some places despite improvements in MDT. Even after treatment, long‐term complications continue, suggesting that elimination goals remain unreached [19]. The immune response to *M. leprae* follows a Th1/Th2 spectrum influencing disease severity [3]. Acute immune reactions and lack of standardized diagnostics underscore the importance of ongoing need for research, effective interventions, and stigma reduction [3].

## 3. Diagnosis and Treatment Outcomes

Although leprosy is treatable with MDT, full recovery is not always possible due to late diagnosis, nerve damage, or treatment complications [26]. Diagnosis primarily relies on clinical signs which can be nonspecific and mimic other skin conditions leading to misdiagnosis [27]. As a result, improving diagnostic accuracy has become a priority. DNA‐based molecular assays developed in recent decades can detect drug‐resistant *M. leprae* directly from clinical samples. Many reference labs in endemic regions can implement these assays providing valuable data for public health strategies and better patient management [9]. PCR is a key tool in this field offering high sensitivity and specificity for *M. leprae* detection especially when clinical signs are ambiguous [28].

Immunological tests like enzyme‐linked immunosorbent assays (ELISAs), which detect antibodies to *M. leprae*‐specific antigens, have also enhanced diagnostic capabilities [29]. Skin biopsy remains the gold standard revealing granulomatous inflammation and acid‐fast bacilli (AFB) in lesions [12]. Additionally, skin tests measuring delayed type hypersensitivity to *M. leprae* antigens help confirm infection [12].

Ridley’s bacterial index (BI) introduced in 1982 classifies patients as MB if BI ≥ 2 at any site or PB if BI < 2 [30]. Alternatively, patients have been classified with purpose of treatment as follows: with more than five lesions are considered MB; those with five or fewer are PB [31].

MDT ensures bactericidal activity against *M. leprae* and was designed to reduce the emergence of drug resistance [9]. MDT combines RIF (rapidly kills bacteria) [1], DDS (inhibits folate synthesis) [32], and CFZ (slowly sterilizes intracellular bacilli) [33].

Alternative therapies including second‐line drugs like fluoroquinolones (e.g., OFL), macrolides (e.g., clarithromycin [CLR]), and minocycline (MIN) target different bacterial pathways [4]. Fluoroquinolones inhibit enzymes crucial for bacterial DNA replication, while macrolides block protein synthesis [34]. These regimens offer solutions for treating drug‐resistant *M. leprae* strains enhancing therapeutic options.

## 4. First‐Line Drugs

The treatment of leprosy has changed over time due to emerging drug resistance. RIF, CFZ, and DDS are the first‐line drugs currently recommended by WHO as the MDT regimen [9]. MDT comes in blister packets with adult and pediatric formulations and is given away for free [31].

### 4.1. DDS

DDS, which was initially introduced as Promin in 1941, became the recommended oral medication by 1947 [1]. This bacteriostatic sulfone prevents *M. leprae* from folate biosynthesis by targeting dihydropteroate synthase (DHPS), encoded by the *folP1* gene [32, 35]. DDS acts as a competitive inhibitor of p‐aminobenzoic acid (PABA), halting bacterial growth. However, DDS resistance has been increasingly reported [36].

### 4.2. RIF

The potent bactericidal drug RIF quickly kills *M. leprae* bacilli. A single 1200‐mg dose can make bacilli undetectable within days [9]. It targets the *β*‐subunit of DNA‐dependent RNA polymerase encoded by the *rpoB* gene [1, 37]. Mutations in *rpoB* reduce drug efficacy by hindering RIF binding [38]. Resistance to RIF presents a serious challenge in treatment stressing the need for combination therapy rather than monotherapy to avoid resistance [39].

### 4.3. CFZ

CFZ, a lipophilic riminophenazine antibiotic developed in the 1950s, is effective in MDT and managing leprosy reactions [40]. It preferentially binds to guanine rich sequences in mycobacterial DNA targeting G + C‐rich genomes [9], known also as B.663 or Lamprene [4]. Though its exact mechanism is not entirely understood, resistance is uncommon and typically associated with mutations in the *Rv0678* gene [4]. CFZ also possesses anti‐inflammatory properties [9]. However, it causes skin pigmentation, darkening both lesions and unaffected areas which may negatively impact patient morale and adherence to therapy [41]. These first‐line medications work well together to treat leprosy, but growing resistance highlights the need for caution and other approaches.

## 5. Other Antileprosy Drugs

### 5.1. OFL

A kind of fluoroquinolone antibiotic increasingly used in leprosy treatment especially when RIF resistance is present [4]. While its exact mechanism is not fully understood, studies have shown it is more effective than DDS and CFZ though still less potent than RIF [4]. Resistance to OFL was first reported in 1994 and has since been observed in other cases [9].

### 5.2. CLR

CLR is a semisynthetic macrolide derived from erythromycin, and it exhibits strong bactericidal effects against *M. leprae* [4]. It works by binding to the 50S ribosomal subunit inhibiting bacterial protein synthesis [4]. A 500‐mg daily dose of CLR kills 99% of *M. leprae* within 28 days and 99.9% within 56 days. Although its mechanism is not fully known, it resembles erythromycin’s mode of action [9].

### 5.3. MIN

MIN is the only tetracycline with significant activity against *M. leprae*, due to its lipophilicity which aids in penetrating the bacterial cell wall [9]. It is bactericidal and more effective than DDS and CFZ, comparable to OFL but less than RIF. Its efficacy increases when combined with RIF and DDS [12]. Resistance is presumed rare given its limited use in single lesion PB cases [9].

New regimens are aimed at shortening treatment durations and improving outcomes. One such combination is ROM (RIF 600 mg, OFL 400 mg, and MIN 100 mg). It has been tested for single lesion PB leprosy [42]. While ROM therapy offers advantages like better compliance and fewer side effects, it provides less protection than WHO‐recommended MDT [4]. Evidence comparing ROM with MDT in MB cases remains insufficient. Continued research is vital to optimize therapies, improve compliance, and resistance [43].

## 6. Evolution of Leprosy Treatment and the Rise of Drug Resistance

The discovery of sulfonamides in the 1940s, particularly DDS in 1948, revolutionized leprosy treatment [4, 44]. However, as DDS is bacteriostatic, lifelong use was required to prevent relapse [4]. By 1959, resistance to DDS was suspected in persistent LL cases mainly due to poor adherence [45]. Confirmed in 1964 following the development of the MFP 191 model [46], resistance soon threatened disease control as resistant strains were transmitted [47]. In 1977, WHO recommended drug resistance testing in unresponsive MB cases using an *in vivo* model [4].

Between 1960 and 1980, RIF and CFZ were added to the treatment arsenal. RIF showed potent bactericidal activity and was widely adopted even in DDS‐resistant cases [4]. However, lack of standardized dosing led to misuse and report of RIF resistance [48].

Meanwhile, MIN, CLR, and OFL emerged as promising second‐line drugs with strong bactericidal activity and minimal side effects, particularly useful in combinations against RIF resistant strains [4, 34]. The recommended regimen includes two second‐line drugs with CFZ for 6 months, followed by one second‐line drug and CFZ for 18 months [31]. Though CFZ resistance is still uncommon, fluoroquinolone resistance has already been reported in areas already affected by DDS and RIF resistance [49].

Efforts to shorten treatment led to the ROM regimen for single lesion PB leprosy [9]. While initially promising, it was less effective than standard MDT and was excluded from recommendations after 2018 due to diagnostic challenges [43].

The WHO also introduced a uniform MDT regimen for both PB and MB patients comprising RIF, DDS, and CFZ [31]. The newest guidelines include a conditional recommendation to modify the antibacterial regimen for treating PB leprosy. Specifically, PB leprosy should continue to be treated for 6 months but with three drugs instead of two. This change aligns the drug combinations used for both PB and MB leprosy. Since the introduction of WHO’s MDT in 1982, PB leprosy has been treated with RIF and DDS for a 6‐month duration, with RIF taken monthly and DDS daily [34, 41].

Though supported by some clinical data [41], concerns arose over side effects especially CFZ‐induced pigmentation leading to stigmatization. One study reported that 9.8% of MB patients discontinued treatment due to pigmentation [41]. As a result, experts urge caution and further research comparing the old and new PB regimens before widespread adoption. Despite concerns, WHO’s latest guidelines endorse the three‐drug regimen for both PB (6 months) and MB (12 months), citing improved outcomes and reduced relapse rates [31].

## 7. Novel Therapies of Leprosy

Despite the success of MDT in reducing leprosy prevalence, its limitations, particularly drug‐related toxicities, adherence issues, and emerging resistance, have prompted the search for alternative strategies. Adverse effects of DDS, including methemoglobinemia, bone marrow suppression, and the potentially fatal dapsone hypersensitivity syndrome (DHS), alongside CFZ‐related skin discoloration and other complications, negatively impact adherence and quality of life [50, 51]. Noncompliance has been identified as a possible contributor to RIF resistance, the cornerstone of MDT efficacy.

To address these challenges, several alternative regimen and novel compounds are being investigated. Regimens such as ROM have shown promise as simplified options [52]. Fluoroquinolones and tetracyclines, particularly OFL and MIN, are bactericidal against *M. leprae*, while newer agents such as rifapentine (RFP), moxifloxacin (MFX), and the diarylquinoline antibiotic bedaquiline (BDQ) demonstrate stronger bactericidal activity [53]. WHO currently endorses fluoroquinolones and MIN in cases of drug‐resistant leprosy, though their long‐term safety requires careful consideration [51].

Recent drug discovery efforts are targeting novel bacterial proteins such as MabA and LipU, offering opportunities for next‐generation therapies [54]. Parallel to therapeutic developments, prophylactic strategies are gaining momentum. WHO now recommends single‐dose RIF postexposure prophylaxis for contacts of untreated patients, while vaccine research including candidates like “LepVax” is aimed at strengthening long‐term prevention [54]. LepVax has been shown to be safe and, in contrast to BCG, it reduces and postpones the neurological complications associated with *M. leprae* infection [55].

Among the novel drugs, BDQ stands out as a particularly promising agent. Originally developed for *Mycobacterium tuberculosis*, BDQ inhibits ATP synthase in *M. leprae*, effectively blocking energy metabolism [56]. Experimental studies show that a single oral dose of BDQ, 3.3 mg/kg, can clear infection in mouse models, with efficacy comparable to RIF and MFX. Its long half‐life and convenient dosing schedule make it especially attractive for improving treatment adherence. Although combination with CFZ did not yield significant additional benefits at tested doses, BDQ is available in both oral and long‐acting injectable formulations (BDQ‐LAIM), the latter offering potential for simplified, less frequent dosing in clinical practice [56].

Emerging evidence highlights the need to revisit standard MDT and transition toward safer, more effective, and adherence‐friendly regimens. New antibiotics, prophylactic strategies, and vaccine candidates collectively represent critical advances toward improving treatment outcomes and reducing transmission of leprosy.

## 8. Leprosy Prevention Through Chemoprophylaxis

Although the exact transmission route of leprosy remains uncertain, close contacts of patients are at higher risk of infection [57]. The disease develops slowly often starting with an asymptomatic phase before progressing to PB or MB forms (over 2–20 years) [12]. Chemoprophylaxis during this early stage can help prevent transmission [4]. First introduced in the 1960s using DDS [58], chemoprophylaxis has evolved to include more effective regimens like single‐dose RIF and combinations with MFX [4].

In 2018, the WHO recommended single‐dose RIF postexposure prophylaxis for close contacts. However, its use has raised concerns about potential RIF resistance in *M. tuberculosis* [4]. Despite this, single‐dose RIF as postexposure prophylaxis has been adopted in several endemic countries [4, 58].

While chemotherapy has cured millions, misuse has led to drug resistance even in emerging genes. Therefore, expanding drug resistance surveillance alongside postexposure prophylaxis implementation is essential for effective leprosy control [4].

## 9. Drug‐Resistant Mechanisms of *M. leprae*

Drug resistance poses a major challenge to global leprosy elimination particularly in high‐burden countries such as Brazil, India, and regions of Southeast Asia [11]. Resistance in *M. leprae* primarily arises from genetic mutations affecting antibiotic target sites complicating effective treatment [6].

RIF resistance, a growing concern, is largely due to missense mutations in the RIF resistance determining region of the *rpoB* gene. The most frequent mutation occurs at codon Ser456, though variations at other codons can also reduce drug efficacy by altering the target binding site [9]. DDS resistance results from mutations in the *folP1* gene, particularly at codons 53 and 55 in its drug resistance determining region (DRDR), which encodes the DHPS enzyme [9]. The first DDS‐resistant strain was reported in 1964, with resistance later linked to extensive monotherapy use [1]. OFL resistance is mainly caused by mutations in the *gyrA* gene, which encodes subunit A of DNA gyrase. These mutations reduce the drug’s binding affinity to its target [4].

In addition to target site mutations, efflux pumps such as ATP‐binding cassette (ABC) transporters and the Mmpl family actively expel drugs, lowering intracellular antibiotic concentrations [59]. These pumps contribute to resistance against RIF, DDS, and OFL. Moreover, the presence of mycolic acid in the cell wall reduces its permeability, limiting antibiotic penetration and further decreasing drug effectiveness in *M. leprae* [59]. Target modification due to mutations in genes like *rpoB*, *ctpC*, and *ctpI* has also contributed to reducing susceptibility [10]. Hence, molecular resistance mechanisms are essential to developing effective therapies against resistant *M. leprae* strains.

## 10. Strategies for Drug Resistance Management

Managing drug resistance in leprosy requires a multifaceted approach. WHO recommends treating RIF‐resistant *M. leprae* with two second‐line drugs, OFL, CLR, or MIN for 24 months, regardless of DDS resistance. If both RIF and OFL resistance are present, CLR, MIN, and CFZ are used [4]. DDS‐only resistance allows continued MB‐MDT, with close posttreatment monitoring [31]. If detected midtreatment, DDS may be replaced with a second‐line drug, though efficacy remains unclear (Table 1) [4, 34].

**Table 1:** WHO treatment guidelines for drug‐resistant leprosy [4, 34].

	**Rifampicin resistance ± dapsone resistance**	**Rifampicin resistance ± dapsone resistance (if intolerant to minocycline)**	**Rifampicin resistance ± ofloxacin resistance**
First 6 months (daily)	Ofloxacin 400 mg + minocycline 100 mg + clofazimine 50 mg	Ofloxacin 400 mg + clarithromycin 500 mg + clofazimine 50 mg	Clarithromycin 500 mg + minocycline 100 mg + clofazimine 50 mg
Next 18 months (daily)	Ofloxacin 400 mg or minocycline 100 mg + clofazimine 50 mg	Ofloxacin 400 mg + clofazimine 50 mg	Clarithromycin 500 mg or minocycline 100 mg + clofazimine 50 mg

## 11. Monitoring Drug Resistance in Leprosy

Systematic monitoring is essential because clinical signs of treatment failure usually become evident only after prolonged therapy, often 12 months or more, and resistance to DDS can be masked by the bactericidal effect of RIF [60].

Traditional phenotypic testing relied on the MFP assay, which allowed evaluation of drug activity but required over a year to obtain results, large quantities of viable bacilli, and significant resources [14]. Other early methods, such as radiorespirometry, were useful for experimental drug discovery but unsuitable for routine clinical use due to dependence on viable bacilli and radioactive substrates [9]. Because *M. leprae* cannot be cultured *in vitro*, classical susceptibility testing remains unfeasible.

The introduction of molecular biology in the late 1990s transformed resistance monitoring by enabling DNA‐based detection of mutations in DRDRs. Currently, the WHO recommends molecular methods targeting rpoB (RIF), folP1 (DDS), and gyrA (fluoroquinolones) [4, 61]. PCR amplification followed by direct DNA sequencing has reliably identified mutations in these genes, and most of the sequenced mutants have been further validated using the MFP assay [62].

Several platforms are now used worldwide: PCR with Sanger sequencing of DRDRs, endorsed by WHO in 2008, and still considered the global reference standard [63]; commercial line‐probe assays, such as GenoType LepraeDR, which are validated for use in reference laboratories and can be implemented in endemic countries [64]; real‐time PCR with high‐resolution melt (HRM) analysis and other probe‐based methods that allow rapid, cost‐effective screening for known mutations; next‐generation sequencing (NGS) and whole‐genome sequencing (WGS), increasingly applied in research and surveillance programs, providing comprehensive mutation profiles and enabling discovery of novel resistance markers [65]. These molecular approaches have now replaced the MFP assay for routine monitoring and have been incorporated into WHO global surveillance networks.

Direct sequencing of PCR amplicons spanning DRDRs is robust for detecting resistance, but its application is often limited in developing countries where leprosy remains prevalent. To overcome this challenge, a simpler technique leprosy drug susceptibility DNA microarray (LDS‐DA) has been developed. This method allows detection of resistance‐associated mutations and facilitates drug susceptibility testing in laboratories closer to field settings, improving accessibility and timeliness of results [15].

Despite these advances, major challenges remain. Validated molecular markers are currently limited to RIF, DDS, and fluoroquinolones, leaving drugs such as CFZ, MIN, and CLR without standardized molecular resistance assays [66]. Access to molecular testing is uneven across endemic countries due to cost, technical expertise, and laboratory capacity. Furthermore, treatment options for confirmed MDR leprosy are limited, with most regimens being empirical and based on repurposed antibiotics or expert consensus rather than large clinical trials. Development of alternative drug combinations, validation of additional molecular markers, and expansion of accessible laboratory platforms are urgently needed to strengthen global leprosy control.

## 12. Leprosy Multidrug‐Resistant (MDR) Cases Worldwide

The emergence of MDR *M. leprae* has become a growing global concern. A 2024 systematic review analyzing global data estimated the overall prevalence of any drug resistance at 11.7% (95% CI: 7.7%–17.3%), with MDR to both RIF and DDS detected in 2.2% (95% CI: 1.2%–3.9%) of cases [67]. Resistance to RIF, the cornerstone bactericidal drug in MDT, is particularly alarming. A meta‐analysis reported a pooled global incidence of RIF resistance of 11% (95% CI: 7%–15%), with rates in relapse cases almost double those in new cases, highlighting the potential role of resistant strains in treatment failure and recurrence [67]. Regional disparities were also evident, with the Western Pacific Region (WPR) showing the highest RIF resistance at 21%, compared to just 4% in the Americas [67].

According to WHO antimicrobial resistance surveillance, data reported from 23 countries including Brazil, Kiribati, Madagascar, Mozambique, and Nepal showed that out of 2109 leprosy cases tested (996 new and 1113 retreatment), resistance to RIF was identified in 27 patients (1 new, 26 retreatment), DDS resistance in 23 patients (3 new, 20 retreatment), and combined RIF‐DDS resistance in 4 retreatment cases [68].

Together, these findings underscore the urgent need for strengthened global surveillance, the development of novel therapeutic options, and region‐specific strategies to curb the spread of resistant *M. leprae* strains.

## 13. Current Global Status of Leprosy

The WHO’s 2023 global update on leprosy highlighted a broad increase in country‐level reporting, with data collected from 184 out of 221 countries and territories across all six WHO regions [69]. This marks a continued upward trend in international reporting coverage. Moreover, this ongoing transmission highlights the challenges in eliminating leprosy. Specifically, leprosy data for 2023 were submitted by 45 countries in the African Region (AFR), 43 in the Region of the Americas (AMR), 22 in the Eastern Mediterranean Region (EMR), 36 in the European Region (EUR), 11 in the Southeast Asia Region (SEAR), and 27 in the WPR. All 23 countries identified as global priorities for leprosy control also submitted reports [69].

According to the most recent global leprosy report published by the WHO, a total of 182,815 new leprosy cases were reported worldwide in 2023, corresponding to a new case detection rate of 22.7 per million population [69]. This represents a 5% increase compared to 2022, during which 174,087 new cases were recorded [70]. Notably, the regions such as AMR, SEAR, and WPR showed a considerable rise in new case detection compared to the figures reported in 2022 (Table 2) [69].

**Table 2:** Comparison of number of new case detection and new case detection rate per million population between 2022 and 2023 [69].

	**Number of new cases detected**		**New case detection rate (per million population)**	
	**2022**	**2023**	**2022**	**2023**
African Region	22,022	21,043	18.5	17.2
Region of Americas	21,398	24,773	20.6	23.7
Eastern Mediterranean Region	3770	2829	4.6	3.4
European Region	55	37	0.1	0
Southeast Asia Region	124,377	131,425	60.1	63
Western Pacific Region	2465	2708	1.3	1.4
World	174,087	182,815	21.8	22.7

Drug resistance data from China suggest that primary resistance to first‐line drugs exists but remains relatively uncommon. In Shandong Province, analysis of 85 isolates of *M. leprae* showed mutation rates in the DRDRs of folP1 (DDS) 1.5%, rpoB (RIF) 8.8%, and gyrA (OFL) 25.9%. Notably, three MDR strains were found even among new cases, one with folP1 + rpoB mutations, two others with rpoB + gyrA mutations [71]. Further surveillance in Zhejiang Province from 2018 to 2021 found 5.9% of isolates with resistance to DDS or OFL, while resistance in other genes (compensatory or less well characterized) was rare and mostly polymorphic [72].

In Japan, molecular genetic analyses of MB leprosy patients since 2000 revealed high rates of mutations associated with drug resistance: folP (52.8%), rpoB (39.4%), and gyrA (16.8%) among studied cases. Some patients had dual or triple mutations (e.g., folP + rpoB, folP + gyrA, or all three), indicating the presence of MDR bacilli [73].

In Indonesia and the Philippines, resistance is lower but measurable, particularly in relapse cases. A study spanning new and relapsed cases across Indonesia, Philippines, and Myanmar found among new cases 3% DDS resistance and 2% RIF resistance. In relapsed patients, the rates were higher: 15% with DDS resistance and 8% for RIF [74]. No quinolone resistance was detected in that study for new or relapse cases there [74].

## 14. Epidemiological Trends in Sri Lanka

Sri Lanka achieved the WHO’s leprosy elimination target in 1995, largely due to the introduction of MDT and an innovative social marketing campaign that raised public awareness and improved treatment uptake [65]. However, despite this milestone, the country continues to report around 2000 new cases annually, leading WHO to classify Sri Lanka as a high‐burden country in the region. Epidemiological data from 1985 to 2021 reveal several important trends. The proportion of child cases declined from nearly 20% in the mid‐1980s to about 10% by the late 1990s and has since remained stable, indicating persistent transmission among younger age groups. Grade 2 disability (G2D) at diagnosis has gradually declined from 10% to 7%, suggesting improvements in early detection, though delays in diagnosis remain. Notably, the proportion of MB cases has increased steadily over the last three decades, reaching 64% by 2021, reflecting a shift toward older patients and long incubation forms of the disease. Geographically, cases are concentrated in districts such as Colombo, Kalutara, Gampaha, Kurunegala, and Batticaloa. More recently, disruptions from the COVID‐19 pandemic caused sharp declines in new case detection, highlighting vulnerabilities in surveillance. These patterns underscore the need for sustained awareness programs, targeted case‐finding, and strengthened contact tracing to interrupt ongoing transmission [65].

## 15. Gaps and Future Directions

*M. leprae* remains a major public health concern particularly in endemic areas with limited healthcare infrastructure. The rise of drug‐resistant strains presents a significant challenge to effective treatment and control [9]. Understanding the mechanisms of drug resistance is critical for developing targeted therapies and preventing further resistance. However, gaps remain particularly in drug susceptibility testing methods like the MFP assay, which are labor‐intensive and may not reflect clinical isolates accurately [1].

Comprehensive genomic studies on resistant *M. leprae* strains are limited [75], impeding a full understanding of resistance genetics and potential drug targets. Additionally, the epidemiology of drug‐resistant leprosy is poorly characterized with most studies region specific and lacking broader context. Socioeconomic factors, treatment adherence, and transmission dynamics must be considered for more effective strategies. Furthermore, chemoprophylaxis programs for contacts need to be enhanced and the effectiveness of newer drugs need to be evaluated [1].

Addressing these challenges requires a multidisciplinary approach involving microbiology, genomics, epidemiology, and clinical research. Key priorities include developing rapid molecular diagnostics and point‐of‐care tests clarifying resistance mechanisms and conducting clinical trials to assess new treatment regimens and identify novel therapeutic targets.

## 16. Outcomes

Although MDT remains the standard treatment regimen, the growing number of MDT resistant cases has highlighted the urgent need for alternative therapeutic options. In response to drug resistance, several substitute drugs have been identified, with many targeting specific mechanisms that contribute to resistance. Molecular research investigations play a pivotal role in understanding these targeting mechanisms facilitating the development of novel drug and more personalized treatment approaches.

## 17. Conclusion

Understanding drug resistance mechanisms in *M. leprae* is vital for effective leprosy treatment and control. Resistance to key drugs like RIF and DDS is primarily caused by genetic mutations in drug target genes such as *folP1* and *rpoB*. Additional factors like efflux pumps, reduced cell wall permeability, and target modifications further contribute to resistance. While MDT, DDS, RIF, and CFZ remain the standard, alternatives like fluoroquinolones, macrolides, and MIN are used for resistant cases. Strengthening efforts in advancing molecular research is vital not only for overcoming drug resistance but also for accelerating leprosy elimination and mitigating the disease’s social stigma and public health burden.

## Disclosure

All authors read and approved the final manuscript.

## Conflicts of Interest

The authors declare no conflicts of interest.

## Author Contributions

G.P., M.T., I.A., and S.S. prepared the first draft of the manuscript. P.C.F., S.S., N.U., and B.D. guided on preparing the manuscript and critically reviewing and revising the manuscript. G.P. and M.T. contributed equally to this work.

## Funding

No funding was received for this manuscript.

## Data Availability

The authors have nothing to report.
